# The variability of isokinetic ankle strength is different in healthy older men and women

**DOI:** 10.1016/j.clinsp.2022.100125

**Published:** 2022-10-31

**Authors:** Felipe Marrese Bersotti, Luis Mochizuki, Guilherme Carlos Brech, André Luiz de Seixas Soares, Jose Maria Soares-Junior, Edmund Chada Baracat, Julia Maria D'Andrea Greve, Angelica Castilho Alonso

**Affiliations:** a School of Arts Sciences and Humanities, Universidade de São Paulo, São Paulo, SP, Brazil; b Graduate Program in Aging Sciences, Universidade São Judas Tadeu, São Paulo, SP, Brazil; c Department of Orthopedics and Traumatology, Faculdade de Medicina da Universidade de São Paulo, São Paulo, SP, Brazil; d Discipline of Gynecology, Department of Obstetrics and Gynecology, Hospital das Clínicas da Faculdade de Medicina da USP (FMUSP), São Paulo, SP, Brazil

**Keywords:** Isokinetic, Muscle Strength, Ankle, Sex, Aged

## Abstract

**Context:**

In the elderly, weak lower limb muscles impair functional tasks' performance.

**Objective:**

To evaluate the healthy elderly's ankle dorsiflexion and plantarflexion maximum torque and its variability in two sets of 5 RM isokinetics evaluation.

**Method:**

50 women (68.0 ± 4.6 years old) and 50 men (72.7 ± 8.5 years old) did two sets of ankle plantar flexor and dorsiflexor isokinetic tests at 30°/s. Peak torque, total work, and coefficient of variation were analyzed.

**Results:**

Men did the strongest plantarflexion torque (*p* < 0.05) and dorsiflexion torque (*p* < 0.05); their highest peak torque occurred at set 2 (*p* < 0.05), while the largest plantarflexion torque variability (*p* < 0.05), dorsiflexion torque variability (*p* < 0.05), and the largest plantarflexion torque variability occurred at set 1 (*p* < 0.05). Men did the highest plantarflexion and dorsiflexion total work (*p* < 0.05) at set 2 (*p* < 0.05).

**Conclusion:**

Older men are stronger than older women. The torque variability, in men, was higher during the first set, suggesting an adaptation to the isokinetics evaluation. Clinicians and researchers should consider that different muscles might need different numbers of sets and trials to measure their maximal muscle strength.

## Highlights

• Older men are stronger than older women.
• Higher torque variability in older men.
• Adaptation to the isokinetics evaluation.
• Different muscles need different numbers of sets.
• Different muscles need different numbers of trials.

## Introduction

Motor variability is observed in trial-to-trial repetitions, during performance, development, and aging, and is inherent to human behavior.^1^ Variations in motor patterns (any peripheral movement representation, such as joint and limb kinematics and kinetics, or surface electromyography) result from more degrees of freedom than equations to solve body movements. More than that, cognition and perception of how to solve problems are individually driven, therefore, solutions to such problems are diverse. Variations in performance come from different physical, motor and physiological conditions. This inherent variability to solve motor problems is an advantage to overcome new^2^ or unexpected situations, but it is severely constrained by disease,^3^ injury,^4^ and aging. Under such circumstances, persons are not able to act like they were without the health condition; their movement patterns are stereotyped.^3^ While such inherent variability in performance shows how important individuality is to achieving internal resources. Testing motor variability enables us to understand two important human features: the ability to adapt and the ability to perform. In general, these two abilities are independent. How aging can affect motor variability? How can we test these two abilities in the elderly? In this study, we are going to apply a variation of a simple strength evaluation to test the hypothesis that aging makes adaptation and performance become associated.

Aging is a natural process involving biological, functional, psychological, and sociological changes, decreasing physical capacities, and slowing down some fundamental physiological processes.^5^ These biological changes impair the capacity for adaptation, and functional reserve, and puts at risk the functional independence. For example, weaker lower limbs (up to 50% at 90 years of age) impair the performance of functional tasks.^6^^,^^7^ Sedentary older adults are weaker^8^ and their movement variability is lower^1^ than young adults. Although movement variability is positive in daily life, movement variability in physical capacity tests is not welcome because it suggests a not trustable system under pressure. Thus, athletes show low variability when they perform maximal physical capacity tests.^9^ It is not clear how variable the physical capacities of elderly persons are.^5^ Although lower limb strength decreases in the elderly, other studies were not concerned about how variable the results were. Why is it important to understand the variability in maximal tests? Because unexpected changes in maximal tests could be a consequence not only of the ability to perform but the ability to adapt. In daily life tasks, maximal force or effort is barely achieved, however, to be confident to do a task is to trust in its own capabilities to perform. If the limits of self-capabilities are not reliable, wrong judgments might occur during motor problem-solving situations. Could it explain the accidental falls in the elderly? Foot and ankle muscle strength affect walking speed,^10^^,^^11^ going up and down stairs,^12^^,^^13^ sitting and supporting itself,^14^ remaining still and balanced.^7^^,^^15^^,^^16^ Accidental falls occur in different situations, but they are usually related to locomotion issues, such as gait initiation, obstacle clearance, or dealing with balance perturbations. To deal with obstacle clearance, the proper lower limb movements demand coordination and proper judgment of clear kinematics. Unreliable force capacity might compromise the obstacle clearance, leading to a fall. The first step to testing such a hypothesis is to understand how variable physical capacities are in the elderly.

Isokinetics evaluations have two aims: evaluate muscle strength and power. Muscle strength is assessed by setting the isokinetics machine to move in low angular velocities (usually below 100°/s); while muscle power is evaluated when the isokinetics is set to higher angular velocities (more than 120°/s). Older adults have problems generating powerful muscle contractions because aging leads to the atrophy of type II muscle fibers.^17^ For this reason, for the elderly, isokinetics evaluations are usually done at low angular speeds. The authors intend to evaluate how elderly people perform 5 Maximal Repetitions (5RM) at a slow angular speed. Brech et al.^18^ and Alonso et al.^19^ have found the second set of a 5RM isokinetics test shows higher results compared to the first one, however, still it is not clear why such results. To better understand why two sets of 5RM may not have similar results, the authors are proposing to evaluate, in the elderly, the ankle muscle strength variability during two sets of 5RM.

There are many studies evaluating torque in the elderly, but studies dealing with variability in this population do not exist.

If the differences between sets were due to motor adaptation to the isokinetics assessment, the authors should expect the torque and work variability during the second set would be smaller. Thus, the purpose of this study is to evaluate the variability of maximal dorsiflexion and plantarflexion torque in healthy elderly people. The authors expect the participants to show different mean maximum torque if we compare two sets of maximum repetitions on the isokinetic dynamometer. Also, the authors expect bilateral symmetry to decrease in the second set of evaluations. Unlike young adults, other populations find it more difficult to achieve their real maximum performance. In the elderly, structural deficiencies caused by aging and acquired diseases are limiting factors.

## Method

### Design and participants

This is a cross-sectional observational study, approved by the Ethics Committee of the University of São Paulo (CaPPesq n° 0468/10). Participants were 100 older adults (50 women, 68.0 ± 4.6 years old, and 50 men 72.7 ± 8.5 years old). The inclusion criteria were: preserved cognitive conditions indicated by the Mini-Mental State Examination (MMSE) (20 points for illiterate people 25 points for elderly people with one to four years of schooling 26.5 points for those with five to eight years of schooling 28 points for those with 9 to 11 years of schooling 29 points for those with more than 11 years of schooling);^20^ no lower limb injury in the last six months; no surgery to affect lower limb mobility; no ankle, knee, and hip mobility restrictions; and normal clinical gait, no limp. The exclusion criteria were unable to do the muscle strength test or the physical exam.

### Isokinetic evaluation

**Fig. 1:**
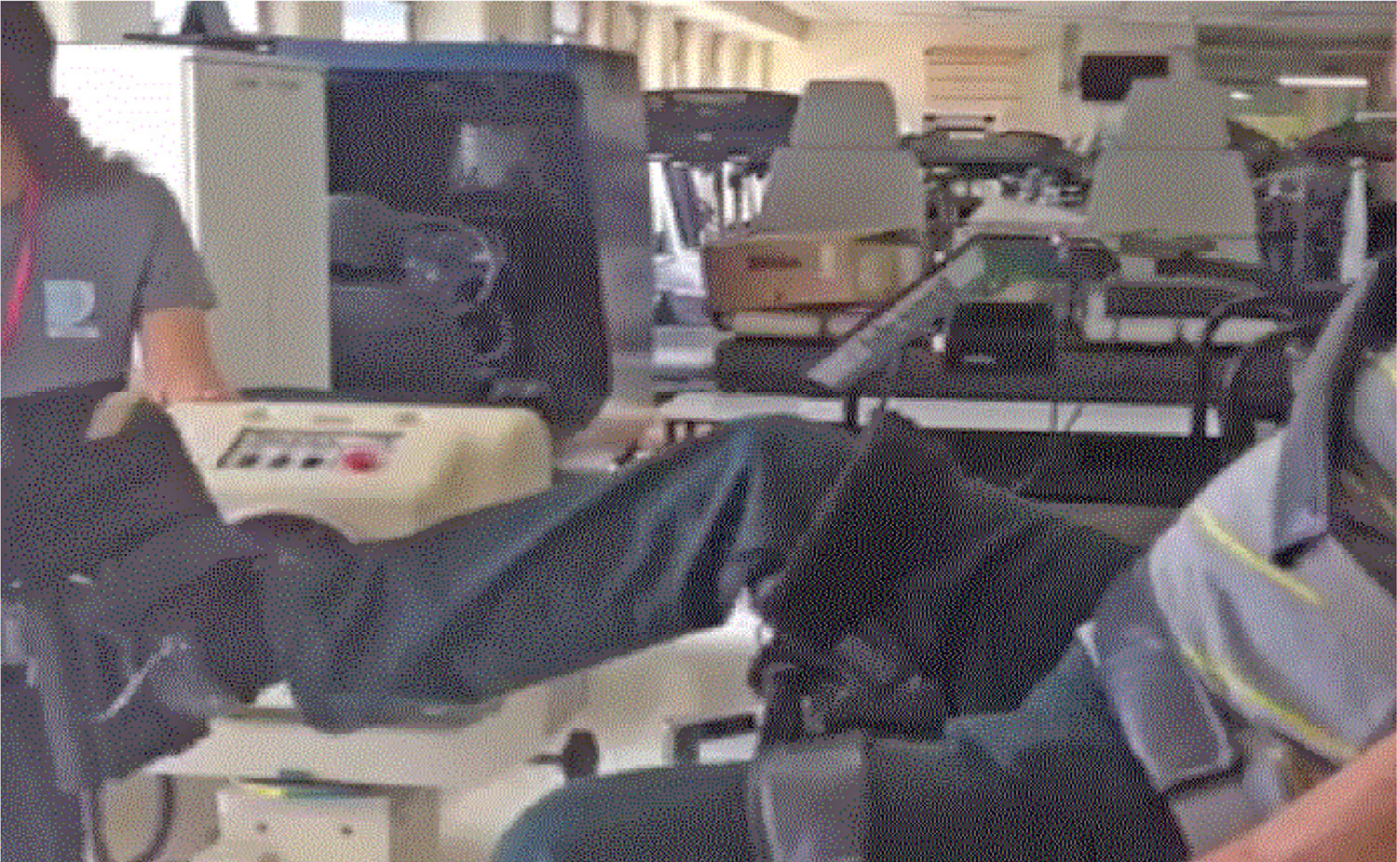
Shows the illustration of the isokinetic evaluation.

Ankle plantarflexion and dorsiflexion isokinetic evaluation (concentric–concentric) was performed (Biodex System 3, Biodex MedicalTM, Shirley, NY, USA). All participants did plantarflexion and dorsiflexion at 30°/s. After the warm-up (three submaximal isokinetic repetitions, i.e., the familiarization trials), the participant sat on the isokinetic chair according to the manufacturer's manual (seated with fasten belt stabilization). The dynamometer axis was aligned with the ankle lateral malleolus, and the torso was positioned at 80° to the hip joint (neutral position 0°). Knee protection was fastened with a strap in the femur's third distal allowing 20° to 30° knee flexion. The examiner ensured each participant's leg was parallel to the floor to avoid the hamstring's action during the test. The participant's active Range of Motion (ROM) was used to define the initial and final movement angles.^9^ Each participant did two sets of Five Repetition Maximum tests (5RM) for the ankle plantarflexion and dorsiflexion of each leg, with 120s rest between sets. First, the dominant leg was tested. These variables were calculated: Peak Torque (PTQ), Total Work (TW), Coefficient of Variation (CV), and agonist/antagonist ratio. Fig. 1 shows the illustration of the isokinetic evaluation.

### Statistical analysis

The Kolmogorov-Smirnov test showed all variables had a normal distribution. Three-way ANOVA (group [men and women], dominance [not dominant and dominant], and set [1 and 2]) was applied to evaluate the ankle strength. The significance level was set at *p* < 0.05. The statistical program SPSS (v.20) was used to run the statistical analysis.

## Results

Three-way ANOVA (group, set, and dominance) showed that group (F_1,394_ = 83.3; *p* < 0.001), and set (F_1,394_ = 10.7; *p* = 0.001) affected peak plantar flexor torque, while there was no effect of dominance. Men had the strongest plantar flexor torque (*p* < 0.05) and the highest plantar flexion peak torque occurred at set 2 (*p* < 0.05). Plantar flexor/dorsiflexor torque ratio was affected by set (F_1,394_ = 7.6; *p* = 0.006). The highest plantar flexor/dorsiflexor torque ratio occurred at set 1 (*p* < 0.05).

Three-way ANOVA (group, set, and dominance) showed group (F_1,394_ = 95.6; *p* < 0.001) dorsiflexor torque, while there were no effects of dominance and set. Men had the strongest dorsiflexor torque (*p* < 0.05). There was no effect of set or dominance

Three-way ANOVA (group, set, and dominance) showed plantarflexion torque variability was affected by group (F_1,394_ = 8.7; *p* = 0.003) and set (F_1,394_ = 13.5; *p* < 0.001), and not affected by dominance. Men had the highest plantar flexor torque variability (*p* < 0.05) and the highest plantar flexor torque variability occurred at set 1 (*p* < 0.05).

Three-way ANOVA (group, set, and dominance) showed that dorsiflexion torque variability was only affected by group (F_1,394_ = 4.5; *p* < 0.001). Men had the largest dorsiflexor torque variability (*p* < 0.05).

Three-way ANOVA (group, set, and dominance) showed that plantarflexion work was affected by group (F_1,394_ = 31.4; *p* < 0.001) and set (F_1,394_ = 6.4; *p* = 0.01), but not affected by dominance. Men had the highest plantar flexor work (*p* < 0.05) and the highest plantar flexor total work occurred at set 2 (*p* < 0.05).

Three-way ANOVA (group, set, and dominance) dorsiflexor total work was only affected by the group (F_1,394_ = 29.9; *p* < 0.001). Men did the highest dorsiflexor total work (*p* < 0.05) (Tables 1, 2, 3 and 4).

**Table 1:** Average and standard deviation of peak torque, total work, and their coefficient of variation (CV) during plantarflexion isokinetic maximal torque at 30°/s.

Group	Side	Session	Peak torque (N.m)	CV (%)	Total work (J)
Women	Dominant	1	37.0 ± 2.8	13.4 ± 1.5	60.3 ± 6.8
		2	42.3 ± 2.8	10.4 ± 1.5	69.4 ± 6.8
	Nondominant	1	38.5 ± 2.8	11.0 ± 1.5	60.2 ± 6.8
		2	42.5 ± 2.8	10.3 ± 1.5	65.8 ± 6.8
Men	Dominant	1	51.4 ± 2.8	17.0 ± 1.5	77.9 ± 6.9
		2	60.7 ± 2.8	11.5 ± 1.5	96.9 ± 6.9
	Nondominant	1	56.7 ± 2.8	17.7 ± 1.5	86.9 ± 6.9
		2	64.2 ± 2.8	11.3 ± 1.5	102.5 ± 6.9

CV, Coefficient of Variation.

**Table 2:** Average and standard deviation of peak torque, total work, and their coefficient of variation (CV) during dorsiflexion isokinetic maximal torque at 30°/s.

Group	Side	Session	Peak torque (N.m)	CV (%)	Total work (J)
Women	Dominant	1	20.8 ± 1.0	6.1 ± 0.9	39.1 ± 2.1
		2	20.6 ± 1.0	5.6 ± 0.9	37.9 ± 2.1
	Nondominant	1	20.9 ± 1.0	6.7 ± 0.9	38.1 ± 2.1
		2	20.6 ± 1.0	5.6 ± 0.9	37.3 ± 2.1
Men	Dominant	1	28.4 ± 1.0	7.8 ± 1.0	46.8 ± 2.1
		2	25.9 ± 1.0	6.8 ± 1.0	43.4 ± 2.1
	Nondominant	1	28.7 ± 1.0	8.7 ± 1.0	48.3 ± 2.1
		2	27.4 ± 1.0	6.6 ± 1.0	46.0 ± 2.1

CV, Coefficient of Variation.

**Table 3:** Mean and standard deviation of ankle amplitude during maximum isokinetic torque in plantarflexion at 30°/s.

Group	Side	Session	Amplitude (in degrees)
Women	Dominant	1	25.00 ± 0.30
		2	25.00 ± 0.32
	Nondominant	1	24.97 ± 0.31
		2	24.95 ± 0.32
Men	Dominant	1	24.83 ± 0.32
		2	24.87 ± 0.27
	nondominant	1	24.84 ± 0.26
		2	24.79 ± 0.34

**Table 4:** Mean and standard deviation of ankle amplitude during maximum isokinetic torque in dorsiflexion at 30°/s.

Group	Side	Session	Amplitude (in degrees)
Women	Dominant	1	25.00 ± 0.30
		2	24.99 ± 0.33
	Nondominant	1	24.97 ± 0.31
		2	24.96 ± 0.31
Men	Dominant	1	24.83 ± 0.32
		2	24.87 ± 0.27
	nondominant	1	24.84 ± 0.26
		2	24.79 ± 0.34

## Discussion

This study revealed older men have stronger plantar flexion and dorsiflexion compared to older women. Moreover, both older women and men did the strongest plantar flexion at the second set of repetitions, but they did not present such behavior for dorsiflexion. These results suggest an adaptation process to develop maximum strength. During this adaptation, the agonist/antagonist ratio decreases from the first to the second set. Therefore, more plantar flexion/dorsiflexion asymmetry in set 2 resulted from more plantar flexion torque and maintenance of dorsiflexion strength. In fact, young and older adults can show a stronger plantar flexor torque compared to dorsiflexion torque.^21^ Moreover, torque variability during the first set was higher than in the second set in men but similar in women. This result supports the present hypothesis about motor adaptation between sets and also suggests older women and men do not have the same adaptation process. Therefore, older women's and men's plantar flexor and dorsiflexor muscles do not exhibit similar behavior during the 5-RM test.

Older men and women have different strength variability. Across five repetitions, older women presented more variation in maximum ankle torque than older men. The greater amount of testosterone hormone^22^, ^23^, ^24^, ^25^ makes men do higher net joint torque than women. However, joint torque and work decrease faster in older men than in same-aged women.^26^ Melzer et al.^24^ found a higher plantarflexion Maximum Voluntary Isometric Contraction (MVIC) compared to dorsiflexion MVIC and they also observed older men were stronger than older women. Webber and Porter^25^ measured the ankle's isometric, isotonic and isokinetic strength in older women twice, with a 7 days interval in between; and they found the plantar flexor torque was greater than the dorsiflexor torque^24^^,^^20^ and only the isometric movement of the plantarflexion torque changed from the first to the second test.

Changing the number of sets and RM in strength evaluations reveals how strong and resistant the elderly muscles are. Two to five RM imply different performances. A strenuous strength test is the 1 RM and it demands long rest intervals to be repeated; the 5RM test is a submaximal strength test, and the 10RM test is indicated for untrained people;^22^ although, in isokinetic evaluations, changing the RM and number of sets may also indicate motor adaptations.^18^ The difference between set 1 and set 2 also raises a reliability issue in strength evaluations,^27^ and the isokinetic RM test in the elderly should be aware of such variability.

Stronger the 5RM performance, the smaller the variability between repetitions. Older men presented the strongest plantarflexion torque in set 2, while the plantarflexion torque had the lowest coefficient of variability. More variability in set 1 suggests the participants were not confident about their limits, or they were saving energy for the next set, although this is true only for the plantar flexor muscles. Greater the torque, the greater its variability. To achieve higher values during the 5RM test, older men also did more total plantar flexion work in set 2. Leyva et al.^26^ showed older men had higher peak torque and work in plantar flexor muscles compared with their dorsiflexor muscles.

Plantar flexion strength behavior changes between the first and second sets. In the first set, the elderly may have explored different strategies to perform the maximal plantar flexion torque, leading to an increase the torque variability; while in the second set, lower variability was combined with the best performance. Therefore, two sets of maximal strength tests may lead to an adaptation process or suggest a learning process, and set 2 is a kind of associative stage when there is less variability in performance, and improvement also occurs more slowly.^28^ The present findings support that the associative stage, in this case, showed greater variability in performance.

This study has limitations. Participants were only tested at just one velocity (30°/s). It was not controlled how physically fit the participants were, although none of the participants was an athlete or highly active. Physical conditions in aging can vary widely due to chronic illnesses. Therefore, the present considerations must be carefully applied if elderly people have health problems. For older adults, clinicians and researchers should consider that different muscles might need different numbers of sets to measure their 5RM performance. The fact that performance between sets was different for plantar flexor muscles should be addressed with covariables such as flexibility, and functional tests.

## Conclusions

In conclusion, the authors found older men were stronger than older women during concentric isokinetics assessments at the ankle joint. Adaptation to isokinetic assessment occurred only in men. Men showed higher plantar flexor and dorsiflexor torques than older women. Torque variability during the first set was higher than in the second set in men but similar in women. Evaluate the variability of maximal dorsiflexion and plantarflexion torque in healthy elderly people, due to this fact, clinicians and researchers should consider that different muscles might need different numbers of sets and trials to measure their maximal muscle strength.

## CRediT authorship contribution statement

**Felipe Marrese Bersotti:** Investigation, Writing – original draft. **Luis Mochizuki:** Formal analysis, Writing – review & editing. **Guilherme Carlos Brech:** Formal analysis, Writing – review & editing. **André Luiz de Seixas Soares:** Investigation, Writing – original draft. **Jose Maria Soares-Junior:** Investigation, Writing – review & editing. **Edmund Chada Baracat:** Investigation, Writing – review & editing. **Julia Maria D'Andrea Greve:** Supervision, Writing – review & editing. **Angelica Castilho Alonso:** Formal analysis, Writing – review & editing.

## Conflicts of interest

The authors declare no conflicts of interest.
